# Vogt–Koyanagi–Harada (VKH)—What Do We Know About the Disease, and Can We Recognize It?

**DOI:** 10.3390/diagnostics16010141

**Published:** 2026-01-01

**Authors:** Maria Boyadzhieva, Preslava Encheva, Dobrin Boyadzhiev, Valeri Sheherov, Darina Koseva, Zornitsa Zlatarova

**Affiliations:** 1 Department of Ophthalmology and Visual Sciences, Medical University of Varna, 9002 Varna, Bulgaria; 2 University Specialized Hospital for Active Treatment in Ophthalmology–Varna, 1606 Sofia, Bulgaria; 3 Department of Optometry and Occupational Diseases, Medical University of Varna, 9002 Varna, Bulgaria

**Keywords:** Vogt–Koyanagi–Harada, syndrome, autoimmune disease

## Abstract

**Background:** Vogt–Koyanagi–Harada (VKH) is a multisystem autoimmune disease that ophthalmologists often encounter first. The condition is caused by an immune response against tyrosinase-related proteins in pigment cells (melanocytes) of the uvea, inner ear, meninges, and skin, and the process may be triggered by genetic and environmental factors. Although much is known about the disease, establishing an accurate and timely diagnosis still requires a multidisciplinary team and strong clinical expertise. Treatment demands early and aggressive anti-inflammatory therapy with corticosteroids, often prolonged and combined with immunosuppressive or biological agents. **Aim:** The present article aims to present three unique cases of patients with VKH syndrome, diagnosed and monitored by Ophthalmologists using standard imaging techniques over the course of five years, to demonstrate the unusual manifestations of the already rare syndrome and to improve the general knowledge of the disease among Ophthalmology specialists. **Methods:** Three different patients with various subjective symptoms and unique clinical signs went through observation in University Specialized Eye Hospital for Active Treatment—Varna. **Results:** The three clinical cases presented diagnostic challenges, the key role of imaging studies and the importance of thorough medical history taking. **Conclusions:** The prognosis in VKH is variable—timely diagnosis and treatment are essential to reduce the risk of recurrence and chronic progression of the disease.

## 1. Introduction

Vogt–Koyanagi–Harada (VKH) syndrome is a rare, autoimmune, multisystemic disorder that primarily targets melanocyte-rich tissues, including retina, meninges, skin and inner ear [1]. The disease is often characterized by a systemic inflammatory response that leads to a range of clinical manifestations, including anterior uveitis, auditory disturbances (such as tinnitus and hypoacusis), alopecia, and skin depigmentation [2].

VKH progresses through distinct stages: the prodromal stage marked by systemic viral-like symptoms; the acute stage which is characterize by bilateral chorioretinitis and panuveitis; the chronic stage that includes pigmentation changes such as vitiligo and the characteristic “sunset glow” fundus; and the recurrent stage, defined by repeating episodes of anterior uveitis and chronic complications like choroidal neovascularization [3,4,5,6,7].

Although the precise pathogenesis of VKH is not fully understood, it is believed to involve a T-cell-mediated autoimmune response against melanocyte-associated antigens with both genetic (e.g., specific HLA-antigens) and environmental factors playing a role [8,9].

Diagnosing VKH requires a high level of clinical suspicion, as its symptoms can overlap with other conditions. The current diagnostic criteria, proposed by the American Uveitis Society, include the absence of eye trauma or surgery, bilateral ocular involvement, specific ophthalmic findings such as choroiditis or sunset glow fundus, neurological symptoms like meningism or cerebrospinal fluid pleocytosis, and skin manifestations such as vitiligo or alopecia [6,10,11].

The treatment primarily involves early and aggressive immunosuppressive therapy to control inflammation and prevent organ damage. First-line treatment typically includes high-dose systemic corticosteroid, followed by a tapering regimen, with the addition of immunosuppressive agents such as Azathioprine or Cyclosporine for long-term management [6,12,13]. In refractory cases, biologic agents such as anti-TNFα and anti-IL-6 may be used [14,15]. Surgical intervention is reserved for complications such as cataract or macular holes [16]. Early and accurate diagnosis is critical to prevent irreversible organ damage.

The presented case reports highlight the clinical course, diagnostic challenges and treatment outcomes of three patients diagnosed with VKH at the University Specialized Hospital for Active Treatment in Ophthalmology—Varna, aiming to illustrate the variability in disease presentation and management strategies. Although Vogt–Koyanagi–Harada disease is a well-described entity, its early diagnosis remains challenging due to heterogeneous initial presentations and incomplete diagnostic criteria at disease onset. This case series aims to highlight common diagnostic pitfalls, illustrate atypical clinical presentations across different age groups, and demonstrate how therapeutic decisions evolve in real-life clinical settings. By presenting three contrasting cases, we aim to provide practical insights into early recognition, monitoring, and escalation of therapy in VKH.

## 2. Materials and Methods

This study is designed as a descriptive, retrospective analysis of three non-consecutive patients diagnosed with Vogt–Koyanagi–Harada (VKH) syndrome at the University Specialized Hospital for Active Treatment in Ophthalmology—Varna. Although ten more cases of patinets with VKH were diagnosed and treated at the institution during the period, we chose these three because of their atypical presentation. The cases were identified through a systematic review of patient medical records over a five-year period, from 2019 to 2024. The aim was to document the clinical course, diagnostic challenges, and treatment outcomes across different presentations of VKH observed in routine clinical practice.

Patients were included if they met the Revised Diagnostic Criteria for VKH or demonstrated a clinical picture highly suggestive of the disease based on ophthalmic, neurologic, or dermatologic manifestations. Only cases with complete documentation of clinical examinations, imaging studies, laboratory tests, treatment regimen, and follow-up were analyzed. Patients with insufficient records, a history of ocular trauma or prior ocular surgery that could interfere with diagnostic interpretation, or confirmed alternative aetiologies for posterior uveitis were not considered for inclusion.

The analysis incorporated data from initial and follow-up ophthalmological assessments, including best-corrected visual acuity, slit-lamp biomicroscopy, dilated fundus examination, tonometry, and visual field testing. Imaging modalities such as optical coherence tomography (Topcon 3D OCT-2000, Tokyo, Japan and Zeiss Cirrus 500/5000, Jena, Germany), fluorescein angiography, B-scan ultrasonography, and, when performed, MRI were reviewed in detail. Laboratory investigations included complete blood count, biochemical parameters, autoimmune markers, infectious serologies and HLA typing when clinically indicated. Interdisciplinary consultations in rheumatology, neurology, and otorhinolaryngology were included in the diagnostic workup when relevant. Therapeutic decisions, including systemic corticosteroid therapy, pulse steroid treatment, local parabular steroid injections, immunosuppressive agents such as Azathioprine and Cyclosporine, and adjunctive topical therapy, were extracted from treatment records.

Given the descriptive and retrospective nature of the study, several methodological limitations are inherent. The non-consecutive case selection and dependency on complete medical documentation introduce selection bias, potentially limiting the representativeness of the sample. Furthermore, the small number of cases and absence of standardized data collection reduce internal validity and do not allow for causal inference. Therefore, the results reflect individual clinical experiences rather than generalizable conclusions, with the purpose of illustrating the variability and complexity of VKH manifestations.

All patient data were anonymized to preserve confidentiality. The study adhered to institutional ethical standards, and informed consent for publication was obtained from all included patients.

## 3. Results

### 3.1. Clinical Case 1

**Figure 1:**
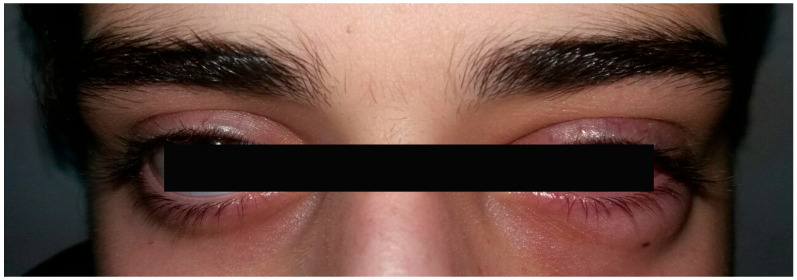
Photograph showing mild eyelid edoema and hyperemia of the left eye, mild conjunctival injection, and pharmacologically induced mydriasis.

A 16-year-old boy presented with complaints of headache and persistent pain localized initially to the left eyebrow and subsequently shifting to the right. At the time of examination, the best-corrected visual acuity (BCVA) was 20/20 in the right eye and markedly reduced to 20/900 in the left eye. Mild conjunctival hyperemia of the left eye was also noted (Figure 1).

**Figure 2:**
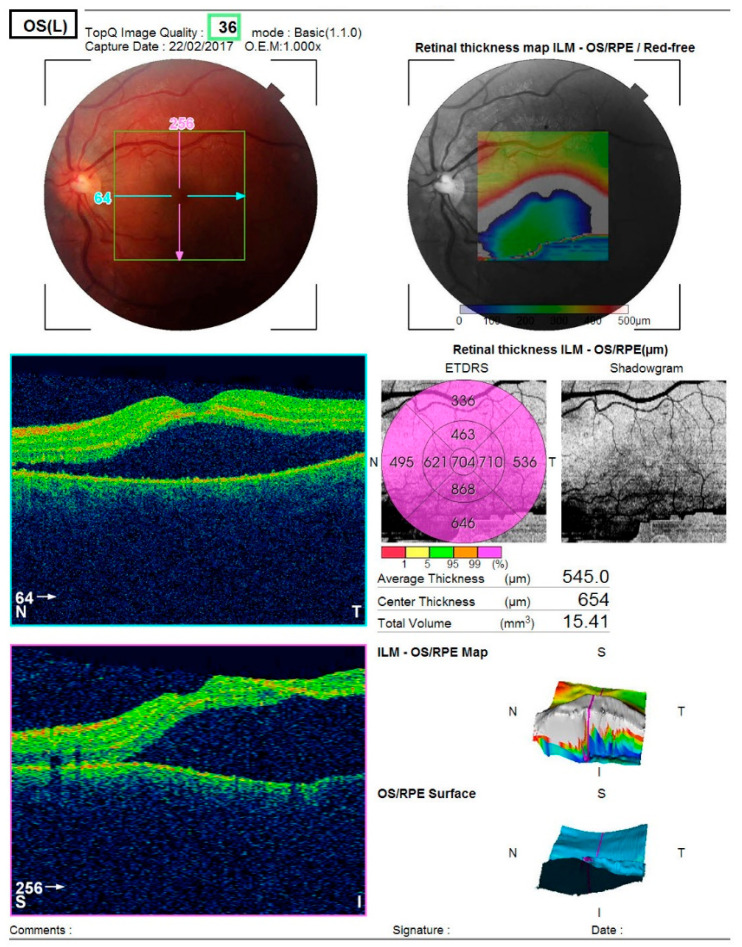
Analysis protocol of optical coherent tomography—Topcon 3D OCT-2000 of the macula of the left eye, showing separation of the neurosensorium from the underlying pigment epithelium with changes in the pigment layer.

Optical coherence tomography (Topcon 3D OCT-2000) of the left macula revealed separation of the neurosensory retina from the retinal pigment epithelium, associated with alterations in the pigment epithelial layer and increased choroidal thickness (Figure 2).

**Figure 3:**
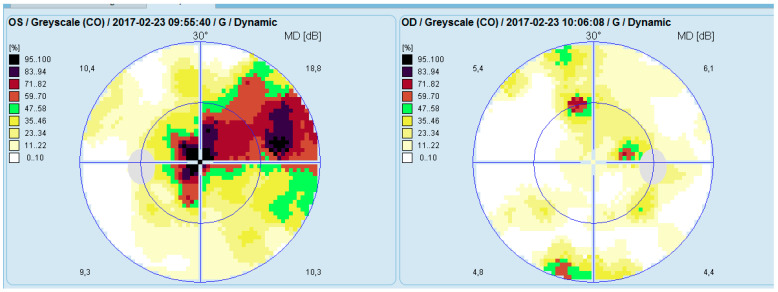
Analysis of a study with Octopus 9000 in the left and right eye.

Computerized perimetry (OCTOPUS 900,Köniz, Switzerland) of the left eye showed an arcuate-like shaped neurofibrillary defect with severely reduced retinal sensitivity originating at the fixation point. The right eye demonstrated several small, localized defects (Figure 3).

Magnetic resonance imaging revealed no abnormalities. A rheumatology consultation and extensive laboratory testing—including complete blood count, biochemical profile, serology for syphilis, antinuclear antibodies, angiotensin-converting enzyme levels, and PPD testing—identified no pathological findings. Lumbar puncture was not performed due to patient refusal. Serology demonstrated IgG positivity for cytomegalovirus (CMV) and herpes simplex virus (HSV), indicating past exposure.

Given the absence of definitive diagnostic criteria at presentation, the marked unilateral visual impairment and the atypical clinical picture, initial management consisted of local corticosteroid therapy with parabulbar Diprophos. This was supplemented with topical corticosteroids, as well as oral therapy with Endotelon to support the vascular wall, in combination with dietary supplements containing lutein, resveratrol, collagen, and hyaluronic acid.

**Figure 4:**
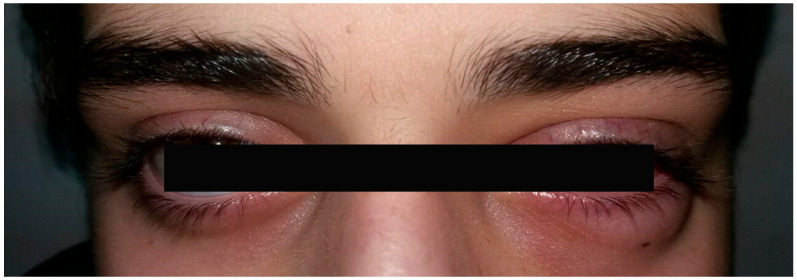
Right and left eye photographs demonstrating injection in the right eye.

Shortly after treatment initiation, conjunctival injection developed in the right eye as well (Figure 4). Bilateral mild anterior uveitis was diagnosed, characterized by fine keratic precipitates, cells and flare in the anterior chamber, and bilateral peripapillary edema, more pronounced in the right eye.

**Figure 5:**
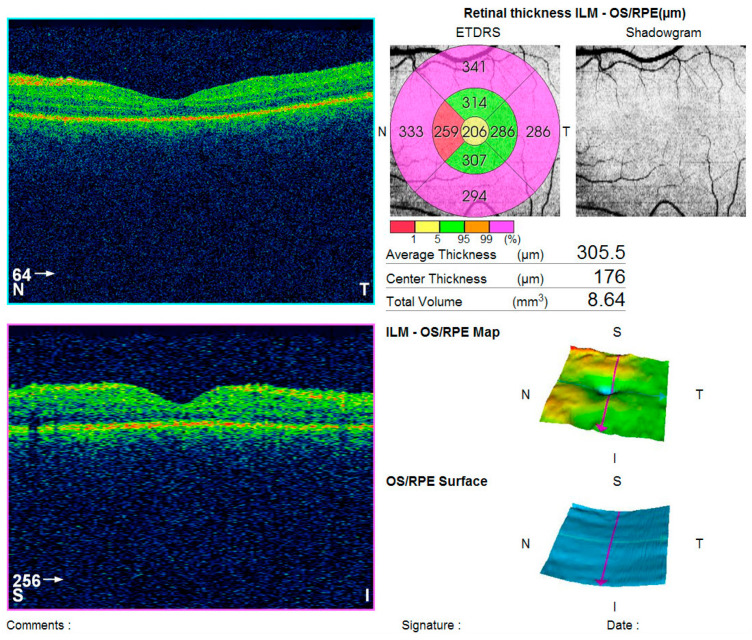
CT (Topcon 3D OCT-2000) of the left macula showing edoema resolution.

**Figure 6:**
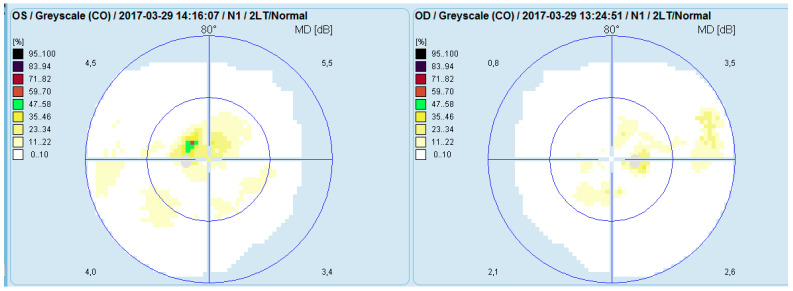
Visual field analysis (Octopus 900) of both eyes following treatment.

One month after treatment initiation, resorption of the retinal edoema in the left eye was observed on OCT (Figure 5), accompanied by improvement in visual acuity and normalization of visual field parameters (Figure 6).

It is important to emphasize that uveitis in children often differs from adult presentations. Pediatric uveitis may be asymptomatic despite significant inflammation and visual impairment, contributing to delayed diagnosis. Additional clinical features—such as optic disk edoema and central serous chorioretinopathy—may further complicate diagnostic precision. Early detection and timely treatment of sight-threatening complications remain essential. Differentiation between VKH and infectious chorioretinitis is particularly critical, given that corticosteroid therapy, essential in VKH, may worsen infectious chorioretinitis by promoting the development of subretinal fibrosis and macular scarring.

Based on the cumulative clinical findings and supportive data from the literature, this case was classified as a probable VKH syndrome. Considering the relatively preserved visual acuity in the presence of bilateral optic disk edoema, a conservative therapeutic strategy was adopted, consisting of close monitoring and local corticosteroid therapy while initially postponing the use of systemic corticosteroids.

### 3.2. Clinical Case 2

A 51-year-old woman presented to our clinic with complaints of markedly reduced vision in both eyes and a persistent headache of several days’ duration. At presentation, the best-corrected visual acuity (BCVA) measured 0.8 in the right eye and 0.04 in the left eye. Slit-lamp biomicroscopy revealed bilateral conjunctival congestion, corneal edoema with diminished transparency, shallow anterior chambers, and an anteriorly displaced irido-lenticular diaphragm. Posterior segment examination of the right eye demonstrated a nasally displaced vascular bundle, whereas evaluation of the left fundus was not possible due to pronounced corneal edoema.

Tonometry using a non-contact tonometer (NIDEK Co. NT-530/510, Gamagori, Japan) showed elevated intraocular pressure (IOP) in both eyes, measuring 35.2 mmHg in the right eye and 42.3 mmHg in the left. Owing to the significantly raised IOP, antiglaucoma therapy was initiated with a fixed combination of Dorzolamide 20 mg/mL and Timolol 5 mg/mL (Duokopt coll.). A working diagnosis of primary angle-closure glaucoma was made, and the patient was scheduled for follow-up examination after four days.

At follow-up, the patient demonstrated further reduction in visual acuity, with BCVA of 0.4 in the right eye and 0.04 in the left. Intraocular pressure had decreased compared to initial measurements, registering 24.8 mmHg in the right eye and 25.6 mmHg in the left; Brimonidine tartrate was added to the antiglaucoma regimen. Preventive YAG-laser iridotomy was performed bilaterally. Despite these interventions, conjunctival injection and a shallow anterior chamber persisted.

**Figure 7:**
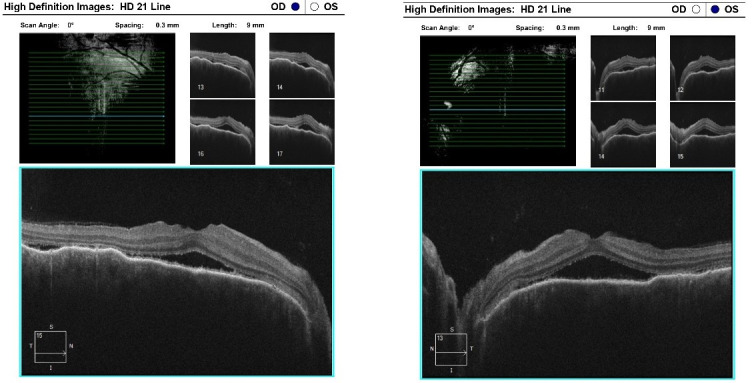
OCT “HD 21 Line” analysis of both eyes showing serous retinal detachment.

Optical coherence tomography (Zeiss Cirrus 500/5000) revealed serous fluid accumulation between the neurosensory retina and the retinal pigment epithelium, resulting in a detachment of these layers (Figure 7). In response, nonsteroidal anti-inflammatory therapy was initiated with topical Yellox drops and oral Vimovo tablets. Given the atypical presentation, a rheumatology consultation was requested, and HLA typing for B5, B27, DR1, and DR4 alleles was performed. Based on these findings, the working diagnosis was revised from primary glaucoma to a probable case of Vogt–Koyanagi–Harada syndrome.

**Figure 8:**
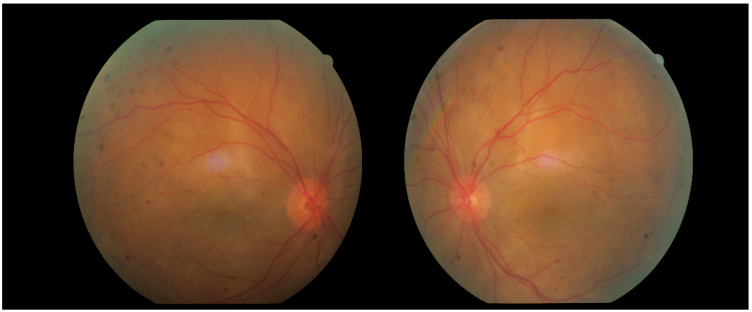
Fundus photographs of both eyes.

**Figure 9:**
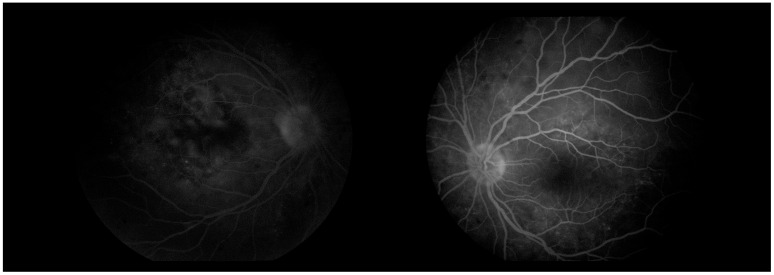
FA (fluorescein angiography).

Three days later, the patient reported a sharp decline in visual acuity, which necessitated a follow-up visit. The visual acuity of the right eye had dropped to 0.04, and in the left eye—she could count fingers in front of the eye. The conjunctiva was bilaterally chemotic, and fine precipitates were observed on the corneal endothelium (Arlt’s triangle). The intraocular pressure was normalized: TOD = 10.8 mmHg, TOS = 12.1 mmHg. From ophthalmoscopy—both maculae had a yellowish reflex, and the exudative retinal detachment in the mid-periphery persisted. From the performed FAG, bilaterally dilated vessels with increased tortuosity, punctate hyperfluorescence in the macula and in the mid-periphery, increasing in intensity and area in the late phases—a sign of edema. (Figure 8) In addition, areas of patchy hypo- and hyperfluorescence are visualized in the mid-periphery bilaterally—areas of exudative retinal detachment. (Figure 9) The patient was referred for emergency hospitalization to initiate intravenous corticosteroid therapy in order to influence the chorioretinal inflammation bilaterally.

**Figure 10:**
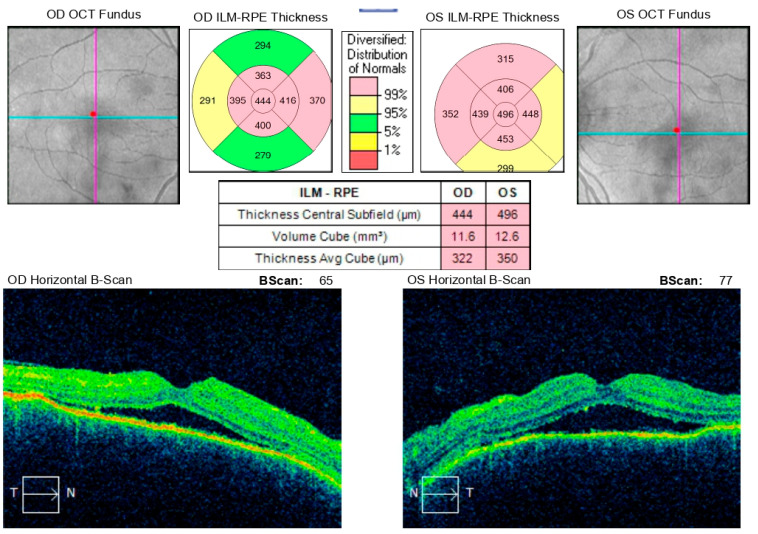
Analysis protocol “Macular cube 512 × 128” of the right and left eye.

During hospitalization, the patient received intravenous pulse therapy with Methylprednisolone. Subsequent assessment demonstrated improvement in visual acuity, with BCVA of 0.25 in the right eye and 0.15 in the left eye, while intraocular pressure was maintained between 12 and 15 mmHg. Follow-up optical coherence tomography revealed mildly blurred optic disk margins bilaterally, evidence of foveoschisis, and partial resolution of exudative retinal detachments (Figure 10). Meanwhile, results from HLA typing became available, which supported the initial suspicion of Vogt–Koyanagi–Harada syndrome.

The patient was discharged with both subjective and objective improvement in visual function and ocular status, characterized by the resolution of conjunctival injection and chemosis, persistence of isolated corneal precipitates, and a deepened anterior chamber. The prescribed home regimen included oral Methylprednisolone (64 mg/day) and topical therapy with Dexanova, Duokopt, Brimogen, and Yellox.

**Figure 11:**
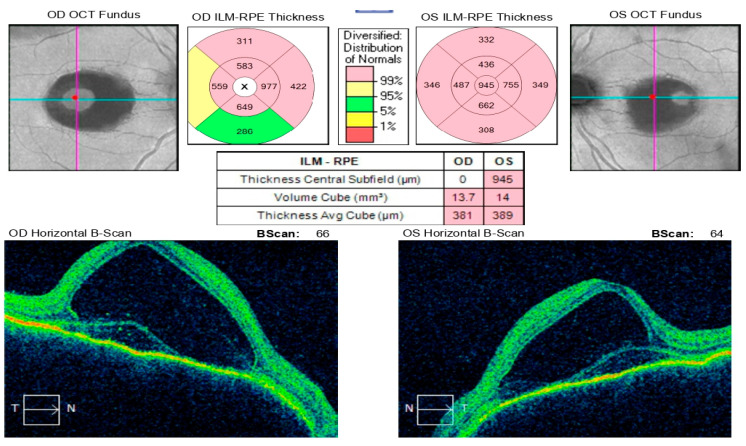
Analysis protocol “Macular cube 512 × 128” of the right and left eye.

At a follow-up visit one week later, the patient reported a decline in visual acuity and persistent headache despite initial improvement following pulse corticosteroid therapy. Examination revealed BCVA of 0.02 in the right eye and counting fingers in the left eye, with bilateral conjunctival injection, corneal edema with endothelial precipitates forming Arlt’s triangle, and a shallow anterior chamber, despite normal intraocular pressure (right eye: 9.8 mmHg, left eye: 12.4 mmHg). Posterior segment evaluation was limited due to corneal opacity. Optical coherence tomography demonstrated macular edema in both eyes and multiple areas of exudative retinal detachment in the mid-periphery (Figure 11). Following multidisciplinary discussion and comprehensive data review, a decision was made to initiate systemic immunosuppressive therapy.

**Figure 12:**
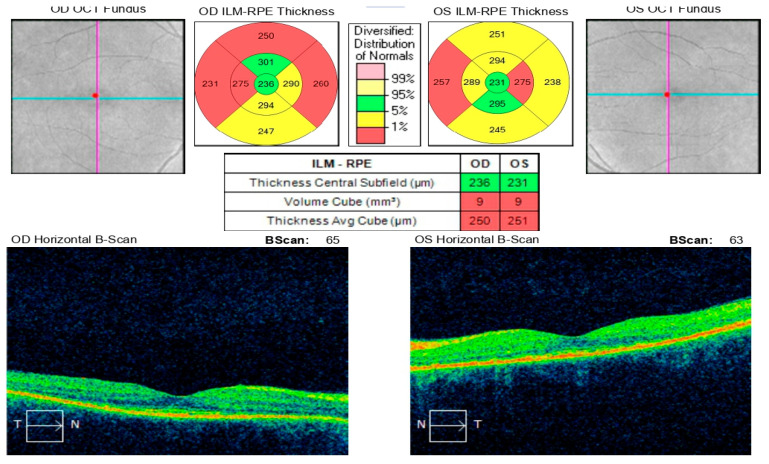
Analysis protocol “Macular cube 512 × 128” of the right and left eye—43 days after the start of immunosuppressive therapy with reverse resorption of edema in both eyes.

A month and a half after initiating treatment with Imuran and Sandimmun Neoral, in combination with oral Methylprednisolone 18 mg/day, the patient returned for follow-up in stable condition. Visual acuity had improved substantially, reaching 0.6 in both eyes. Intraocular pressure was within normal limits, the conjunctiva appeared quiescent, the cornea was smooth and transparent, and the anterior chamber was moderately deep. Fundoscopic examination revealed macular depigmentation in both eyes, without reflex abnormalities (Figure 12). According to the patient’s report, the persistent headache had also resolved following initiation of immunosuppressive therapy.

This case illustrates that, although corticosteroid therapy can provide initial improvement, it may be insufficient to achieve sustained remission or complete resolution of Vogt–Koyanagi–Harada syndrome. Accumulated evidence suggests that early initiation of combined therapy with corticosteroids and immunomodulatory agents—ideally from the onset of disease, even prior to the development of anterior uveitis—can induce durable remission, potentially achieve full disease resolution, and prevent long-term complications.

### 3.3. Clinical Case 3

A 30-year-old woman presented to our clinic with pain localized to the left eye and the left side of the head, while concurrently receiving treatment for otitis media and bronchitis. In the emergency room, she received a single intramuscular injection of Dexamethasone, which provided only transient symptomatic relief. Ophthalmologic examination revealed best-corrected visual acuity of 1.0 in both eyes and intraocular pressure within normal limits (right eye: 19.7 mmHg; left eye: 19.6 mmHg). Biomicroscopic evaluation of the anterior segment demonstrated a normal right eye, whereas the left eye exhibited edematous and hyperemic upper and lower eyelids, tenderness on palpation, and prolapse of the lacrimal gland upon eyelid eversion; the conjunctiva was inflamed, with all other structures appearing unremarkable. Based on these findings, a working diagnosis of dacryoadenitis was established. Local therapy was initiated, including topical administration of a nonsteroidal anti-inflammatory drug, corticosteroid, and antibiotic (Maxitrol and Dicloabak).

**Figure 13:**
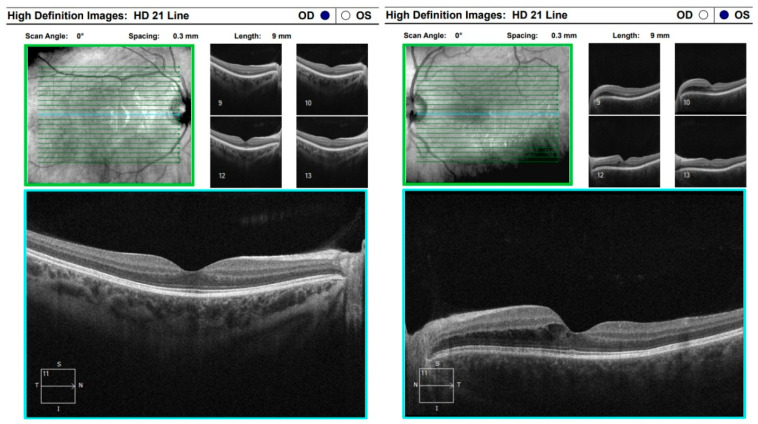
Analysis protocol “HD 21 Line” of the right and left eye.

**Figure 14:**
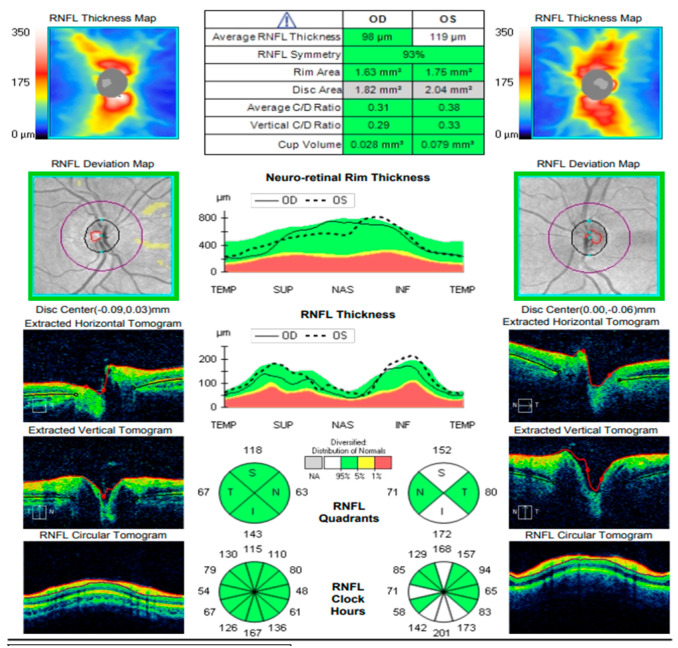
Analysis protocol “Optis Disk Cube 200 × 200” on the right and left eye.

At the scheduled follow-up, although the periocular inflammation had improved, the patient reported markedly blurred vision in the left eye, with BCVA measured at 0.9 and subjective metamorphopsia on the Amsler grid. Biomicroscopy revealed pronounced anisocoria, with the left pupil wider than the right but still reactive to light. Fundoscopic examination was unremarkable in the right eye, while the left eye demonstrated macular edema. Optical coherence tomography (Zeiss Cirrus 5000) confirmed macular edema with isolated cystoid spaces and no evidence of optic disk swelling (Figure 13 and Figure 14). B-scan ultrasonography revealed bilateral scleral thickening, prompting revision of the working diagnosis to probable bilateral scleritis, likely of autoimmune origin. Parabulbar administration of Dexamethasone was performed, resulting in improvement of both subjective symptoms and objective findings.

**Figure 15:**
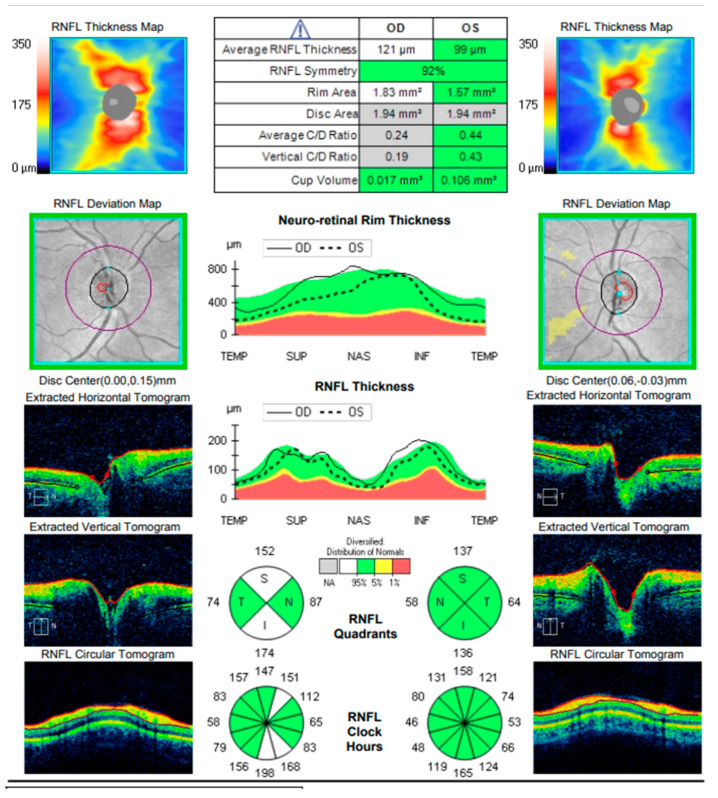
Analysis protocol “Optis Disk Cube 200 × 200” on the right and left eye.

Approximately six weeks later, the patient presented with complaints of transient, painless visual loss. Examination revealed visual acuity of 1.0 in both eyes, however red color perception was impaired in the right eye. Biomicroscopy of the anterior segment was unremarkable in both eyes. Fundoscopic evaluation demonstrated blurred and indistinct optic disk margins in the right eye, consistent with papilledema, while the left eye appeared normal (Figure 15). Given these findings, the patient was admitted urgently with a working diagnosis of optic neuritis, and intravenous pulse corticosteroid therapy was initiated.

Following marked subjective and objective improvement with inpatient therapy, the patient was discharged with a prescription for oral corticosteroid therapy for home use. Subsequent follow-up examinations demonstrated stable ocular status with no abnormalities detected.

**Figure 16:**
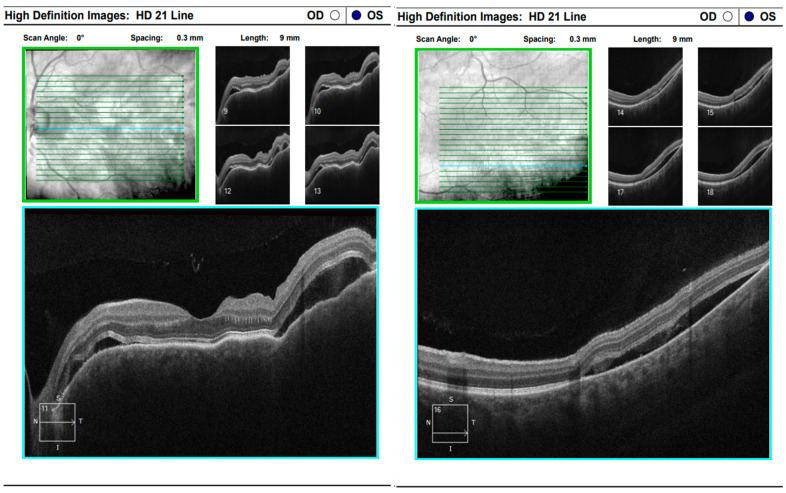
Analysis protocol “HD 21 Line” of the left eye.

One year later, the patient returned to the Eye Hospital—Varna with complaints of pain, discomfort, and difficulty focusing in the left eye, accompanied by persistent headache. Visual acuity in the right eye remained 1.0, with no observable structural abnormalities. In contrast, visual acuity in the left eye had decreased to 0.7, with subjective metamorphopsia. Fundoscopic examination of the left eye revealed an exudative retinal detachment nasal to the macula, which was confirmed by optical coherence tomography. During the evaluation, the patient reported a history of hearing difficulties and presented documentation from an otorhinolaryngology consultation indicating idiopathic sensorineural hearing loss. OCT demonstrated paramacular edema with detachment of the pigment epithelium extending from the papilla to the nasal macula, consistent with chorioretinal inflammation in the left eye (Figure 16).

**Figure 17:**
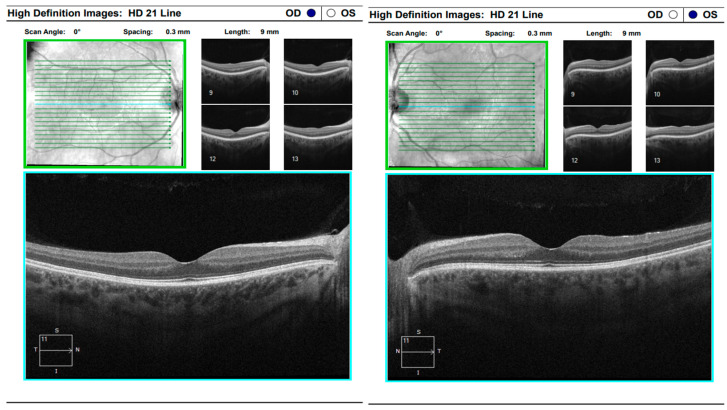
Analysis protocol “HD 21 Line” of the right and left eye.

**Figure 18:**
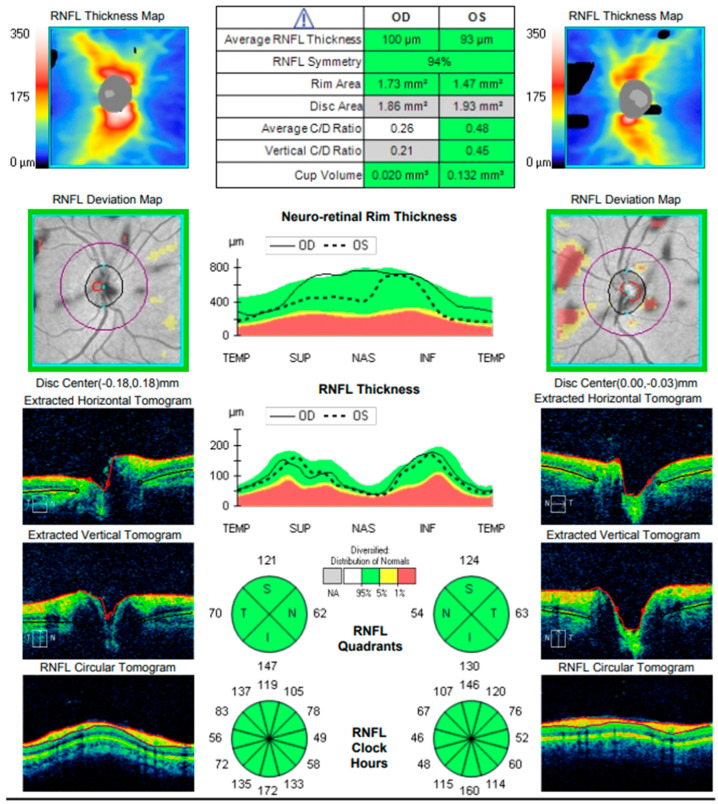
Analysis protocol “Optis Disk Cube 200 × 200” on the right and left eye.

Comprehensive interdisciplinary evaluation of the clinical and imaging data supported a diagnosis of Vogt–Koyanagi–Harada (VKH) disease, prompting urgent hospitalization. Pulse therapy with intravenous Methylprednisolone was initiated, complemented by local parabulbar administration of Dexamethasone. The patient exhibited rapid improvement, with resolution of subjective symptoms, normalization of visual acuity in the left eye to 1.0, and marked reduction in papilledema. She was discharged with a prescription for oral Prednisolone to be continued for a minimum of six months. At nearly one year following the most recent hospitalization, the patient’s condition remains stable (Figure 17 and Figure 18).

This case illustrates the wide spectrum of clinical manifestations associated with Vogt–Koyanagi–Harada (VKH) disease. The diagnostic process may require repeated reassessment, as VKH can mimic several systemic and ocular conditions. A thorough and detailed patient history is essential for accurate diagnosis, and regular follow-up is critical to ensure long-term disease control and timely management of potential complications.

## 4. Discussion

The three cases presented herein underscore the highly variable clinical course of VKH disease and illustrate the diagnostic and therapeutic challenges it poses. Across our cases—spanning ages from adolescence to middle adulthood—VKH manifested through different initial presentations (serous retinal detachment, ocular hypertension, uveitis, optic nerve involvement, chorioretinal inflammation), requiring repeated reassessment and modifications in therapy. These observations echo findings from larger series and real-life studies, which demonstrate that even with prompt therapy, VKH may evolve unpredictably over months to years, often requiring long-term follow-up and individualized management.

Several long-term follow-up studies have documented favorable outcomes when VKH is managed with a combination of systemic corticosteroids and, often, immunosuppressive therapy. For instance, in a cohort of 38 patients followed for a mean period of 120 months, the mean final visual acuity significantly improved compared to baseline; patients treated with initial intravenous corticosteroids experienced fewer relapses, fewer monthly recurrences, and a lower incidence of “sunset-glow fundus,” compared to those managed with oral corticosteroids only [17].

Similarly, a retrospective series involving 26 patients over two decades documented a shift in recent years from steroid monotherapy toward combined immunosuppressive therapy (IMT) plus low-dose steroids. In that series, 81% of patients receiving combined IMT/steroids achieved disease stability at 24 months, with significantly better visual outcomes than those on steroids alone [18].

Moreover, a long-term report of pediatric VKH treated with biologic therapy—specifically Infliximab plus Methotrexate—described 10 years of stable disease without relapses, after tapering off systemic steroids [19]. These data collectively support the notion that early and appropriately sustained immunosuppressive therapy can induce long-term remission, and in many cases may allow discontinuation of systemic steroids.

Conversely, other series have reported chronic or recurrent disease in a sizable proportion of patients: rates ranging between 17.5% and 79% [17]. This underlines that even with aggressive initial therapy, VKH remains prone to recurrence or chronic evolution, especially when immunosuppression is inadequate or tapered too rapidly.

Our experience aligns with the growing consensus: systemic corticosteroids remain the mainstay of initial therapy, but their use alone may be insufficient to guarantee sustained remission or prevent long-term complications. In our second and third cases, despite initial control of inflammation, relapse occurred during tapering, necessitating escalation to immunosuppressive therapy.

The shift toward early combined immunosuppression is well supported: early introduction of agents such as methotrexate, azathioprine, mycophenolate mofetil, or cyclosporine has been linked with higher rates of remission, fewer relapses, reduction in steroid burden, and better visual prognosis [18]. In refractory or chronic recurrent cases, biologic agents—including TNF-α inhibitors such as Adalimumab—have shown promise; a recent study reported good control of intraocular inflammation in refractory VKH with Adalimumab, highlighting its role as second-line therapy when conventional regimens fail [20].

In addition, local therapy (periocular or intravitreal steroids) may serve as adjuncts or steroid-sparing alternatives in selected cases. For example, sustained-release intravitreal dexamethasone implants have been associated with improved BCVA and resolution of macular edoema in relapsing posterior uveitis due to VKH, reducing reliance on systemic steroids [21].

Taken together, these findings suggest a treatment paradigm in which early aggressive therapy—ideally within weeks of symptom onset—is followed by a carefully planned maintenance regimen, with immunosuppressants introduced early or at first signs of relapse. This approach aims not only for control of active inflammation but also for prevention of chronic depigmentation (sunset-glow fundus), chorioretinal atrophy, and other sight-threatening complications.

In our Case 2 and Case 3, initial reliance on corticosteroids (local or systemic) was insufficient to prevent relapse or chronic recurrent inflammation. Only after addition of immunosuppressive therapy did the patients achieve sustained remission and stabilization of visual acuity. This mirrors the literature, where combined therapy tends to yield better long-term control. The relapsing nature and chronicity of VKH in these cases further underscore the need for long-term follow-up and readiness to escalate therapy.

Our Case 1—a probable VKH in a pediatric patient—highlights another important dimension: VKH in younger patients may present atypically, with subtle or delayed anterior segment inflammation, and a high risk of diagnostic delay. This reinforces the idea, noted in pediatric reports, that treatment must be individualized and vigilantly monitored [22].

Although there are limited formal studies evaluating “health-care worker satisfaction” or “health-care burden” in VKH management, the shifting paradigm toward early immunosuppression and long-term maintenance therapy carries important implications. For clinicians—especially in regions where VKH is rare—maintaining high suspicion for VKH, ensuring early referral to uveitis centers, and implementing long-term management plans can improve patient outcomes while reducing the need for repeated interventions, hospitalizations, and high-dose systemic steroid courses. This may translate into improved workload efficiency and better resource utilization in specialized centers.

Moreover, long-term immunosuppressive regimens (as opposed to repeated high-dose steroid pulses) may reduce systemic side-effects in patients and decrease the frequency of acute exacerbations requiring emergent care—thereby potentially improving patient quality of life and reducing the burden on health-care services.

As a case series of three patients, our findings are inherently limited by small sample size and potential selection bias. The retrospective design and non-standardized follow-up schedule may lead to underestimation of subclinical recurrences. Furthermore, in the absence of advanced imaging modalities such as enhanced-depth imaging OCT (EDI-OCT) or indocyanine green angiography (ICGA) in all cases, subtle choroidal inflammation may have gone undetected.

## 5. Conclusions

Vogt–Koyanagi–Harada (VKH) syndrome is a multisystem autoimmune disorder commonly first identified by ophthalmologists. Regular monitoring using fluorescein angiography and optical coherence tomography is critical for the ongoing assessment and management of patients with VKH. Early diagnosis and prompt initiation of treatment are essential to minimize the risk of disease recurrence and to prevent progression to chronicity.

## Data Availability

The raw data supporting the conclusions of this article will be made available by the authors on request.
